# Multidimensional correlation magnetic resonance imaging in low- and middle-income countries: opportunities and barriers to equitable deployment

**DOI:** 10.3389/fpubh.2026.1870188

**Published:** 2026-07-15

**Authors:** Valentine Terfa Genyi, Bamidele Omotayo Awojoyogbe, Michael Oluwaseun Dada, Oyeleke Ismail Olarinoye, Henry Ananyi Sanni

**Affiliations:** 1 Department of Physics, Federal University of Technology, Minna, Niger State, Nigeria; 2 Department of Physics, Federal College of Education, Okene, Kogi State, Nigeria; 3 Department of Nuclear Science, Federal University of Technology, Minna, Niger State, Nigeria

**Keywords:** challenges and benefits, computational techniques, diagnostic imaging, low- and middle-income countries (LMICs), multidimensional correlation MRI, pattern recognition

## Abstract

Multidimensional Correlation Magnetic Resonance Imaging (McMRI) represents a promising advancement in medical imaging, offering enhanced diagnostic accuracy through the integration of multiple MRI parameters into unified datasets. In low- and middle-income countries (LMICs), McMRI could significantly improve early disease detection and treatment planning, particularly for complex conditions such as cancer and neurological disorders. Benefits include richer data acquisition, improved tissue characterization, and the potential for cost-effective, non-invasive diagnostics. However, practical barriers remain: limited infrastructure, scarcity of trained personnel, high implementation costs, unstable power supply, and data governance challenges hinder widespread adoption. Addressing these issues requires efficient computational workflows, sustainable technology investment, and capacity-building initiatives tailored to LMIC contexts. This review highlights both the transformative potential and the practical obstacles of deploying McMRI in resource-constrained settings, underscoring the need for collaborative strategies that align technological innovation with healthcare equity.

## 1 Introduction

Low- and middle-income countries (LMICs) face extensive challenges in healthcare delivery, particularly regarding access to essential imaging modalities and subsequent diagnostic concerns (1). There is limited availability of magnetic resonance imaging (MRI), computed tomography (CT), and ultrasound modalities, while laboratory services frequently require the sending of samples to external facilities due to the absence of laboratories on-site. MRI is globally the least available of established imaging technologies, creating an urgency for alternate approaches (2, 3).

Multidimensional Correlation MRI (McMRI) is an advanced imaging approach that integrates multiple MRI parameters such as relaxation times (T1, T2, T2*), diffusion properties, spectroscopic data, and temporal dynamics into a single correlated framework. Unlike conventional multiparametric MRI (mpMRI), which acquires and interprets each parameter separately, MC-MRI emphasizes the relationships between parameters, capturing how they vary together across tissues and disease states. Thus while mpMRI collects separate datasets (e.g., T_1_, T_2_, diffusion) and interprets them independently, providing complementary but fragmented information, MC-MRI correlates these datasets mathematically, uncovering hidden relationships between parameters and produces integrated, multidimensional maps that enhance diagnostic precision and reduce variability. MC-MRI can improve differentiation between tumors and abscesses, enhance glioma grading, and accelerate acquisition by leveraging correlations rather than requiring repeated scans (4). Those techniques identifies and models correlations between different MRI signals, enabling richer tissue characterization, while correlation-adaptive frameworks are computational models that adjust to the strength and type of correlations present in multidimensional data, improving reconstruction accuracy and diagnostic reliability. When joint multidimensional are incorporated, they represent a unified representation of MRI signals across multiple dimensions (spectral, temporal, spatial), allowing simultaneous analysis rather than isolated parameter evaluation. This makes it particularly valuable in LMIC healthcare systems, where scanner time and resources are limited.

Numerous subjective factors and technically demanding processes give rise to a high degree of variation in routine clinical MRI much higher than that typically observed in other general or advanced imaging modalities resulting in disparities in diagnosis and reporting accuracy, interpretability challenges, and reporting time. Tackling this challenge, multidimensional correlation MRI offers substantial benefits through the regular recording, retrospective reconstruction, and integrated analysis of multi-sequence data (5). Through such integrated processing, the smoothness, stability, and exactitude of reported metrics are markedly enhanced, lowering the burden on clinicians and improving healthcare delivery.

## 2 Conceptual foundations of multidimensional correlation MRI

MRI quantifies compartmentalized water or lipid content to infer the physiological or pathological state of tissues. Compartmentalization can have diverse origins at different spatio-temporal scales. In the human body, these scales range from the microscopic (sub-cellular) distributions of lipid bilayers and associated macromolecular structures; through the multi-cell layer organization in white matter axons or the organization of morphologically identifiable cell types; to intra- and inter-organ arrangements of tissues. Accessing these different scales, and the associated models, constitutes the basis for multidimensional MRI (6, 7).

### 2.1 Theoretical basis of multidimensional correlation

Multidimensional Correlation Magnetic Resonance Imaging (MC-MRI) captures complex relationships between different MRI measurements, such as spectral, temporal, and diffusion signals. Instead of analyzing each measurement in isolation, MC-MRI integrates them to reveal hidden connections between tissue properties and disease patterns (8). By correlating signals across multiple dimensions (e.g., relaxation times, diffusion behavior, tracer dynamics), MC-MRI provides a richer characterization of tissue microstructure. This enables clinicians to distinguish subtle pathological changes that conventional single-parameter MRI might miss (9). For example, combining T_1_ and diffusion correlations can improve the differentiation of tumors from abscesses, while multi-echo correlations enhance the accuracy of tissue relaxation measurements (10).

**Figure 1:**
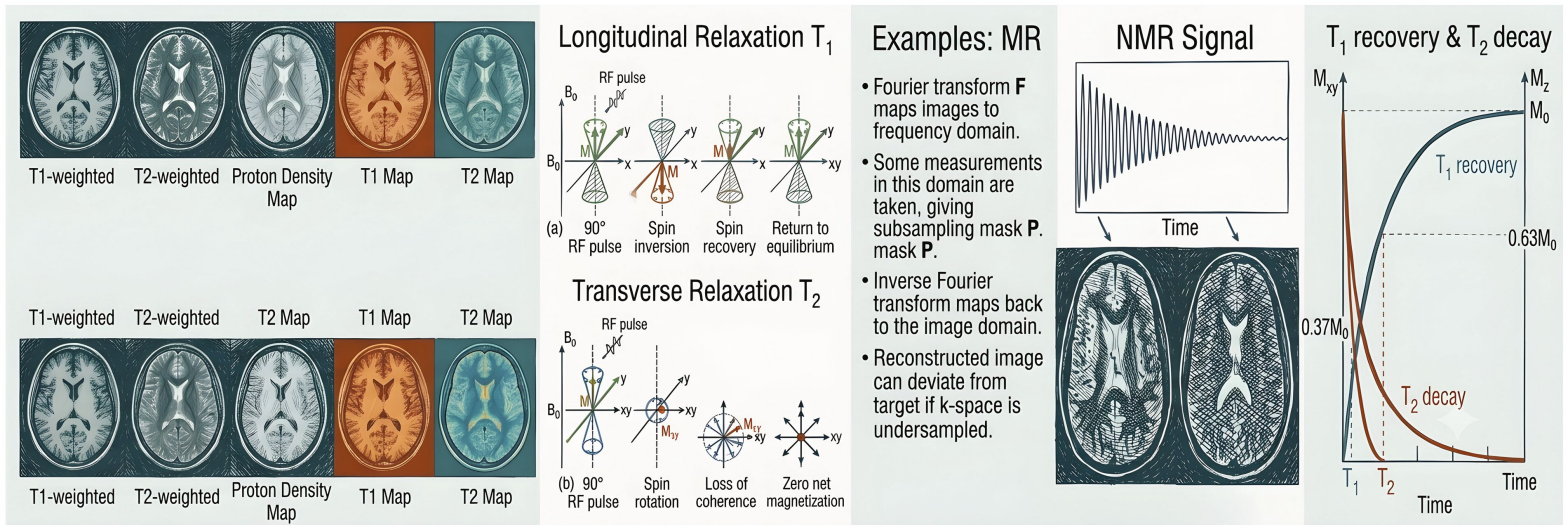
This figure demonstrates how spectroscopic, multi-echo, multi-contrast, and multi-temporal data streams converge into a unified MC-MRI framework. This shows the distinction between conventional mpMRI (separate datasets) and MC-MRI (correlated datasets), showing how integration improves tissue characterization and diagnostic precision (54).

The strength of MC-MRI lies in its ability to move beyond simple signal extraction toward comprehensive tissue characterization. This approach improves diagnostic accuracy, supports personalized treatment strategies, and opens new opportunities for computational innovation (11). To achieve this, robust algorithms and standardized data specifications are required to ensure reliable reconstruction and interpretation of multidimensional datasets. Thus, shifting MRI analysis from simple signal extraction to comprehensive tissue characterization improves clinical decision-making and creates new opportunities for computational innovation (see Figure 1).

To advance scientific understanding of MRI analysis, there is a need for robust algorithms, standardized data specifications, and computational tools that support accurate multidimensional image reconstruction and interpretation. MRI acquisition pathways are diverse and include T_1_ relaxation characterization, diffusion analysis, tracer-based imaging, exchange diffusion studies, water compartment analysis, and saturation recovery measurements, all of which contribute to improved tissue characterization and disease diagnosis.

### 2.2 Computational techniques in MRI

**Figure 2:**
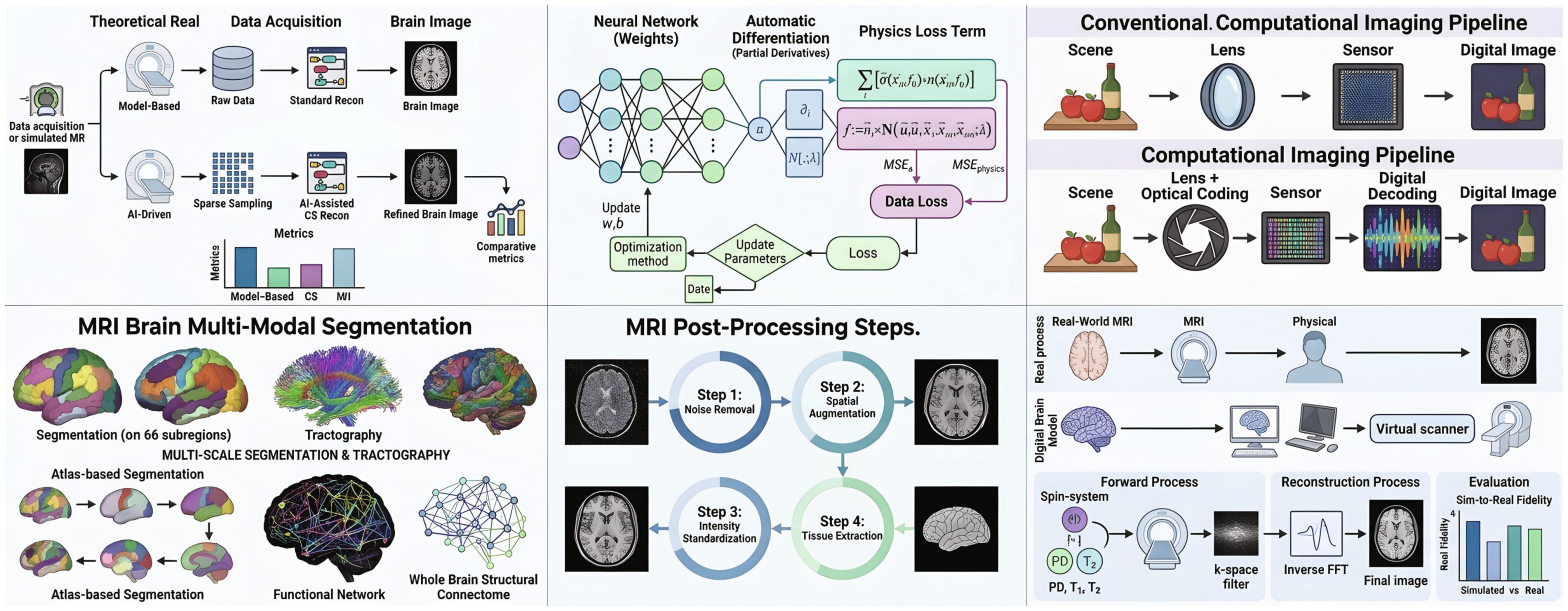
The pipeline illustrates the step-by-step computational workflow: data acquisition → signal processing → kernel construction → inverse problem solving → multidimensional reconstruction. This figure explains how correlation-adaptive frameworks reduce noise, improve reconstruction accuracy, and enable scalable deployment in LMICs (55).

The signals captured by an MRI system can be treated either as a spatially varying function of time (2D or 3D+T data) or as a function of additional variables (e.g., contrast agents), resulting in higher-dimensional datasets. In the former case, the signal originates from the free induction decay (FID) produced by the excited nucleus, while in the latter, the signals represent the temporal evolution of the corresponding image. MRI signals vary in response to changes in electromagnetic fields, spatial gradients, and temporal acquisition parameters. These variations provide important information for image reconstruction and tissue characterization. Therefore, the MRI signal can be represented as a combination of spatial and temporal basis functions that describe the underlying tissue properties (see Figure 2).

Various computational techniques used in multidimensional MRI can be categorized into tensor decomposition, statistical modeling, and signal separation methods. Tensor decomposition is an established methodology to model and analyze multidimensional correlation (12). While these methods have demonstrated efficiency and some clinical applications, further development of smoothing filters and onboard reconstruction algorithms is still required.

Recent advances in automated pattern recognition and high-dimensional modeling allow codes and bases to be obtained without prior assumptions about signal changes, enabling integration of different sequences. These approaches support knowledge transfer across multiple imaging modalities, often providing more clinically useful information than single-modality analysis. A major limitation of such computational models is the need for large amounts of high-quality training data, which can be difficult to obtain in clinical MRI settings. Signal separation techniques are well established; however, they often require different algorithms for different signal patterns, which limits their general applicability (13).

## 3 Methodology for literature selection

This manuscript is structured as a narrative review with elements of a scoping review. Literature was identified through searches in PubMed, Scopus, IEEE Xplore, and Google Scholar using combinations of keywords such as “multidimensional correlation MRI,” “multiparametric MRI,” “tensor decomposition MRI,” and “low-field MRI LMIC deployment.” Publications from 2010 to 2025 were prioritized to capture both foundational and recent advances. Inclusion criteria focused on peer-reviewed articles, clinical case studies, and technical reports directly addressing MC-MRI, correlation-adaptive frameworks, or LMIC deployment. Exclusion criteria removed papers limited to general AI discussions without direct MRI application. Screening involved title/abstract review followed by full-text assessment to ensure methodological rigor and relevance. Supplementary sources such as WHO reports and LMIC case studies were included where peer-reviewed data was limited. This methodology yielded a curated set of references that balance technical foundations, diagnostic applications, and LMIC case studies, ensuring coherence and focus on MC-MRI.

### 3.1 Relevance to low- and middle-income countries

Multidimensional Correlation MRI offers substantial opportunities for improving health services in Low- and Middle-Income Countries (LMICs). LMICs experience a dramatic growth in Magnetic Resonance Imaging (MRI) demand, influenced by rising disease burdens (including neurological, neoplastic, and cardiovascular pathologies), population growth and aging, and improving accessibility to MRI technology (2, 14, 15). MRI is among the top three technologies requested in many LMICs, yet, at the same time, access to MRI remains severely limited: fewer than 10% of countries have MRI facilities, and only 2% of LMICs have established national MRI networks (3). MRI capabilities are constrained by scarcity of MRI machines, limited access to spare parts, monthly operating costs exceeding the GDP per capita, lack of formal national or international training programs, unavailability of professional societies, time consuming and cumbersome acquisition sequences, and lack of knowledge transfer from high to low-income countries. Within this context, addressing access barriers that disproportionately affect LMICs aligns directly with global health priorities articulated by the World Health Organization and the United Nations Sustainable Development Goals.

MRI data acquisition can proceed along multiple physical dimensions that characterize particular signal properties such as space, time, and frequency. Such data can be further aggregated through mathematical correlation, yielding a joint projection of the different involved data modalities. The resulting multidimensional correlation MRI combines information from multiple attribute dimensions, extracts new dimensions with clinical relevance, and addresses open MR data and disease detection and characterization challenges. Addressing multiple factors is particularly salient in LMICs, with limited data available for overcoming the ubiquitous non-representativeness and non-diversity of financial, spatiotemporal, and epidemiological gradients. Such gradients expand even further when multiple sites are considered, and systematic disparities between local data and datasets generated in wealthier regions weaken the performance, implementation, and dissemination of advanced MR accessibility approaches.

### 3.2 Healthcare needs and MRI access

A significant global disparity persists in MRI availability, with units often regarded as costly and inaccessible in low- and middle-income countries (LMICs). The operation of conventional MRI systems requires highly trained personnel, stable infrastructure, and substantial financial resources, making them difficult to sustain in resource-constrained settings.

Recent innovations in sustainable MRI technology including cryogen-free magnets, lightweight designs, and advanced reconstruction software offer more affordable alternatives without compromising diagnostic performance. Low-field MRI systems, in particular, provide a viable option for LMIC healthcare ecosystems by reducing acquisition costs, lowering maintenance demands, and minimizing environmental impact (3).

Investing in these scalable technologies, alongside workforce training and infrastructure support, is critical to expanding diagnostic imaging capacity in LMICs. Such strategies can help bridge the gap in access while ensuring that MRI contributes meaningfully to equitable healthcare delivery (16).

### 3.3 Data diversity and representativeness

Low- and middle-income countries (LMICs) face persistent challenges in establishing MRI capacity due to limited resources and uneven global distribution of installations, with the majority concentrated in high-income regions (3, 17). This imbalance amplifies healthcare disparities and restricts access to advanced diagnostic imaging. The complexity of MRI data highlights the importance of computational techniques that can enhance diagnostic accuracy and efficiency, particularly where trained experts are scarce. Multidimensional Correlation MRI, by integrating temporal, spectral, and spatial dimensions, offers richer diagnostic information and faster interpretation. However, its operational success in LMICs depends on careful evaluation of scalability, accessibility, and generalizability.

The complexity of multidimensional MRI data presents interpretive challenges, but also yields richer diagnostic information than single-parameter approaches. Non-invasive processing pipelines enable integration of routine imaging datasets without requiring repeated patient scanning, supporting more consistent clinical decision-making while reducing the burden on limited resources. In LMIC contexts where scanner access is restricted, these pipelines maximise the diagnostic value of each acquired dataset by enabling retrospective multi-parameter analysis from a single acquisition session (18).

## 4 Computational techniques: benefits for LMICs

The Multidimensional Correlation Imaging (MDCI) workflow can output a variety of multidimensional imaging data from MRI signals, potentially addressing pressing clinical needs in LMICs (19, 20). The workflow begins with the acquisition of raw MRI data, followed by a series of processing steps tailored to the chosen imaging modality. A signal model is first fitted to capture essential spatial and spectral dimensions. From this, low-dimensional feature descriptors are extracted, denoised, and correlated across multimodal MRI datasets and clinical metadata. Multidimensional images are then reconstructed, after which the data undergoes advanced post-processing, such as classification through computational modeling and statistical analysis techniques. MDCI methods can improve operational efficiency by enhancing multimodal magnetic resonance spectroscopic imaging (MRSI), quantitative contrast enhanced imaging, and diffusion MRI, while also supporting correlation with clinical metadata from other imaging sessions.

### 4.1 Acceleration and efficiency

Health systems in LMICs face major challenges due to the burden of diseases such as tuberculosis, cancer, and trauma (14). Although MRI can provide crucial insights, the full potential of the technology is often not realized because of the limited availability of scanning equipment and the predominance of repetitive, low-dimension data. Consequently, there are multiple needs for MRI provision in LMICs: the capacity to undertake diverse scans, the capability to transfer acquired data to specialized distant diagnostic centres, and the functionality to extract additional information from existing data already collected but not yet interpreted.

Computational approaches designed for multidimensional data help address these needs. By operating on multiple dimensions, such as signals acquired through time or space, these methods enable greater interpretation of the data collected. Frameworks that rely on correlation mathematical definitions of inter-dependence across dimensions can significantly accelerate MRI acquisition and analysis. This gain in efficiency specifically benefits LMIC environments where resources are limited but health outcomes remain a high priority (21).

### 4.2 Enhanced diagnostics through multidimensional data

Enhanced diagnostics can be attained by adopting rapid cardiovascular magnetic resonance (CMR) protocols that streamline modalities to lower costs and complexity. Ultrafast CMR scanning has been validated to gauge disease burden in LMICs, with acquisition times of 5 min or less (1, 22). Short protocols for assessing cardiac function and scarring are being implemented in-country, altering patient management and demonstrating sustainability across multiple centers. Supporting local manufacturing of MRI components, including superconducting magnets and scanners, enhances affordability and accessibility, aided by governmental and private sector initiatives (16). Investment in training and research, coupled with promotion of international collaboration, is essential for developing more appropriate imaging solutions and cultivating local expertise. Open-access dissemination of technology blueprints and software further stimulates domestic innovation, curbs maintenance expenses, and mitigates global disparities in radiological service provision.

### 4.3 Resource optimization and telemedicine prospects

Expanding access to MRI in low- and middle-income countries (LMICs) requires strategies that reduce scan times and leverage remote diagnostic support. Demand for imaging consistently exceeds the limited number of available scanners, with LMICs carrying a significant share of the global disease burden but operating only a small fraction of MRI units.

Multidimensional Correlation MRI can help optimize resources by accelerating acquisition, improving reconstruction, and enabling remote interpretation. Telemedicine platforms further extend these benefits, allowing off-site collaboration and diagnostic support across both urban and rural settings. By combining faster imaging protocols with scalable telehealth solutions, McMRI has the potential to strengthen diagnostic capacity in resource-constrained environments, ensuring more equitable access to advanced imaging technologies (22).

## 5 Computational challenges and limitations

**Figure 3:**
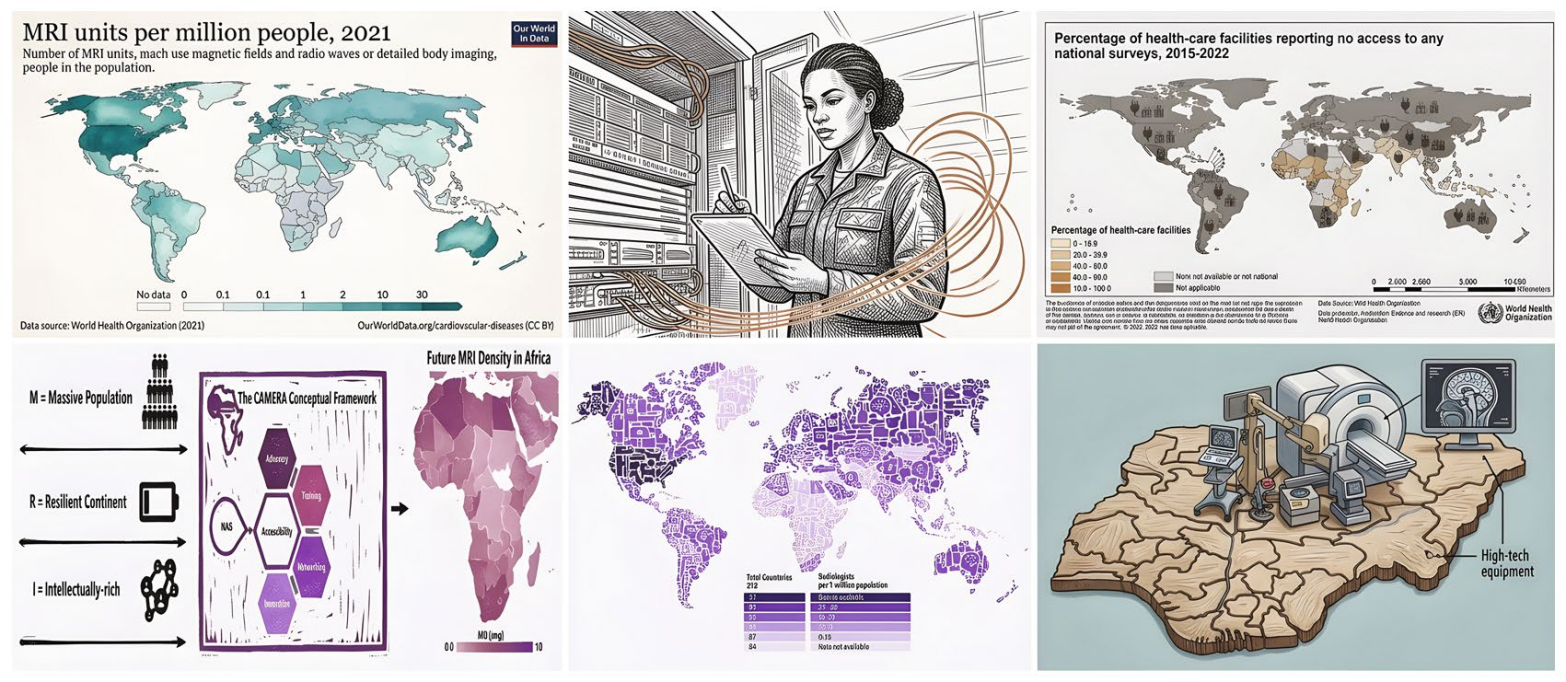
This figure visually summarizes barriers such as limited MRI availability, high computational costs, poor infrastructure, and workforce shortages. This LMIC section emphasize how challenges directly affect MC-MRI adoption, and sets the stage for presenting country-specific case studies as practical solutions (56). (Original schematic, authors’ own work).

Multidimensional correlation magnetic resonance imaging (MRI) in low- and middle-income countries (LMICs) has the potential to enhance detection and diagnosis by exploiting correlations among image data acquired at different times or through different modalities. Although positive results have emerged from other health domains, computational approaches that facilitate data fusion can pose significant barriers. In LMICs, they can accelerate scanner access and utilization; shorten workflow, reconstructions, or acquisition times; improve diagnostic accuracy; assist interpretation; and enable telemedicine or remote reading. However, the associated computational challenges need careful consideration; if left unaddressed, they can compromise the benefits (see Figure 3).

Major computational challenges include corruption or degradation of imaging data during storage, transfer, and analysis; lack of standardization in acquisition parameters; incompatibility between scanners from different manufacturers; incomplete metadata; infrastructural limitations such as unstable electricity supply; limited access to repair services; and the proprietary nature of many computational algorithms and distributed platforms (23).

### 5.1 Data quality and standardization

The widespread adoption and use of diffusion MRI (dMRI) for probing the microstructural architecture of the brain has been hindered by a lack of consensus on what constitutes good-quality acquisitions and how data quality affects the extraction, estimation, and interpretation of dMRI-derived indices. Diffusion indices such as fractional anisotropy (FA) and mean diffusivity (MD) are influenced by numerous variables and therefore require caution in interpretation.

Artifacts such as motion, vibration, and field inhomogeneity are significant in multi-center studies, especially when targeting infant populations or individuals with developmental disorders. These challenges call for caution regarding dataset selection and reinforce the need for flexible protocols. Recent work has explored data-driven computational modeling for estimation of diffusion tensor imaging and multi-shell filtration, but reproducibility across diverse cohorts remains a concern (24).

### 5.2 Computational resource constraints

Diagnosing diverse diseases and abnormalities crucially depends on MRI. In LMICs, reinforcing MRI capacity is essential for comprehensive diagnostics. Multidimensional correlation MRI offers great benefits, but limited healthcare access and inequities create major challenges for robustness and trustworthiness.

Computational methods improve diagnostic potential through extraction of tissue-specific features, but they also impose constraints. Ensuring data quality and interoperability remains difficult due to diverse MR equipment and reconstruction pipelines, as well as incomplete metadata. Energy consumption, hardware maintenance, and infrastructural instability further limit computing capabilities (25, 26). Moreover, the opaque nature of certain computational algorithms poses risks for reproducibility and external validation. Careless implementation may reduce clinician trust, underscoring the importance of transparency and standardized evaluation before clinical deployment.

### 5.3 Algorithm transparency and validation

Many imaging algorithms lack transparency, inhibiting independent investigation and obstructing identification of unintended consequences (27). Preprocessing, reconstruction, and post-processing must be meticulously documented to guarantee reproducibility. To foster transparency, open-source implementations should be shared along with algorithms in peer-reviewed manuscripts, permitting validation against reported outcomes. Thorough documentation, including parameter ranges and application-specific details, enhances transparency further.

Algorithm validation is equally critical, encompassing development and independent scrutiny to verify accuracy, robustness, and generalizability (18). Although dedicated validation metrics exist, some medical imaging lacks standard benchmarks or datasets, complicating comparison with state-of-the-art outcomes. Substantial inter- and intra-scanner variability further hampers direct cross-institutional evaluation. Consequently, access to diverse publicly available datasets for independent algorithm development, testing, and validation constitutes a vital enabler for effective implementation of imaging in LMICs (19, 28, 29).

### 5.4 Equity, bias, and generalizability

Tackling equity, bias, and generalizability promotes greater consistency in imaging protocols and supports resource-constrained settings without monopolizing access to acquired data. Less-differentiated information can serve broader populations and expedite deployment of computational imaging methods (30).

Equity considerations and the non-exclusivity of approaches suggest intermediate regulatory frameworks, such as data trusts, collaboration protocols, and co-governance, promoting joint ownership, stewardship, and equitable sharing of data, code, and findings. The uneven distribution of CT and MRI systems across regions reflects general inequities in medical resources. Although improvements have been noted, healthcare imbalances remain regionally pronounced. Further, despite stringent technology regulatory regimes, unlicensed copies circulate widely in LMICs, engendering concern over safety and efficacy (29).

## 6 Summary of key studies on multidimensional correlation MRI in LMICs

**Table:** 

Study/source	Focus area	Key contribution	Relevance to LMICs
Superpixel-Guided Graph-Attention Boundary GAN for Adaptive Feature Refinement in Scribble-Supervised Medical Image Segmentation.	Multimodal medical image analysis	Demonstrated integrated computational approaches for improved diagnostic precision and efficiency	Shows feasibility of correlation-based MRI to maximize diagnostic yield with limited resources
Cross-Attention Patch Fusion for Few-Shot Colorectal Tissue Generation	Workflow optimization	Highlighted scalable, infrastructure-aware deployment strategies	Confirms that resource-constrained environments can adopt advanced imaging workflows
Low-Field MRI Innovations (19, 28, 29)	Sustainable imaging technology	Introduced cryogen-free magnets and lightweight scanner designs	Offers cost-effective solutions for LMICs with limited budgets and unstable infrastructure
Global telemedicine implementation and integration within health systems to fight the COVID-19 pandemic: a call to action (31)	Remote diagnostics	Explored off-site acquisition, reconstruction, and interpretation	Extends diagnostic reach to rural and underserved areas
Equity and Governance Frameworks (30)	Data governance and bias mitigation	Proposed collaborative data trusts and co-governance models	Ensures equitable access and safeguards data sovereignty in LMIC deployments
Ultrafast CMR Protocols	Rapid cardiovascular MRI	Validated short acquisition times (<5 min) for cardiac function and scarring	Demonstrates sustainable, time-efficient imaging suitable for LMIC hospitals
Open-Source Algorithm Validation (18, 19, 28)	Transparency and reproducibility	Advocated for open-source implementations and standardized validation metrics	Builds clinician trust and supports reproducibility in diverse LMIC settings
Workforce Training & Local Manufacturing	Capacity building	Highlighted importance of training programs and domestic production of MRI components	Reduces reliance on imports, lowers costs, and strengthens local expertise

### 6.1 Implementation pathways in LMIC contexts

MRI in low-and-middle-income countries (LMICs) has become crucial under the WHO Universal Health Coverage agenda. Many ‘silent’ brain and vascular diseases have now surged due to the epidemiological transitions from infectious to chronic diseases as well as unhealthy lifestyles that prevail in LMICs, leading in turn to the highest increase of years lived with disability (YLDs) in low-income groups and countries. The limited MRI availability remains a significant barrier to consultations, scans, interpretations, and even preventive measures. Multidimensional correlation MRI is of great interest in LMIC settings, where routine low-field MRI scanners and screening sequences are deployed to cope with affordability and sustainability requirements (20). Notably, it has enabled low-cost diffusion-tensor-like and arterial-equivalent perfusion with sub-millions of dollars’ scanner and turnkey system.

### 6.2 Infrastructure and capabilities

In low- and middle-income countries (LMICs), limited MRI infrastructure restricts the ability to deliver timely and accurate diagnoses. Conventional one-dimensional MRI modalities capture only a single acquisition parameter, which narrows the scope of diagnostic information. By contrast, integrating multiple MRI modalities within a unified computational framework enables more comprehensive tissue characterization and supports earlier detection of pathological changes.

Multidimensional Correlation MRI addresses this need by combining diverse imaging parameters into correlated datasets, producing richer diagnostic insights without requiring repeated scans. This approach enhances interpretability, reduces variability, and strengthens clinical decision-making. For LMICs, where resources are limited, such integration offers a practical pathway to maximize the utility of existing imaging systems while advancing diagnostic capacity (32, 33) (see Table 1).

**Table 1:** Table showing the real-world implementations pathways.

Country	Challenge highlighted	Implementation pathway	Citation
Nigeria	Scarcity of high-field MRI, unstable electricity, limited maintenance	Introduction of low-field and portable MRI units in university hospitals; solar-powered backup systems to stabilize power supply; workforce training for MRI technicians	(41, 60–63)
Uganda	Late cancer detection, shortage of advanced scanners	Use of ultrafast DCE-MRI for early breast cancer detection; Kisubi Hospital’s acquisition of Siemens Magnetom Flow Plus with helium-independent cooling and AI-powered workflows	(64, 65)
India	High costs of high-field MRI, rural accessibility gaps	Deployment of 0.4 T open low-field MRI in rural hospitals for spine/back pain diagnosis; portable low-field MRI prototypes for bedside and ICU imaging	(66, 67)
South Africa	Workforce shortages, safety compliance gaps	Pilot studies on MRI safety training in public hospitals; University of Cape Town initiatives on stroke imaging and workforce development	(68, 69)

These case studies provide concrete pathways for implementation, moving beyond generic descriptions and illustrating how infrastructure limitations, workforce shortages, and maintenance barriers can be practically addressed in LMIC contexts.

### 6.3 Capacity building and training

Multidimensional Correlation MRI (McMRI) represents an emerging methodology for the analysis of multimodal MRI data. Despite its potential, the integration of McMRI into clinical workflows in low- and middle-income countries (LMICs) has not yet been systematically investigated (59). Assessing its applicability in these contexts requires consideration of the diagnostic and monitoring relevance of current McMRI acquisitions, the design of datasets optimized for clinical utility, and the computational strategies capable of supporting their analysis. Advancing this line of inquiry not only enables novel scientific insights but also contributes to capacity building by equipping national personnel with expertise in advanced data and signal processing techniques, thereby fostering sustainable improvements in healthcare delivery (2).

MRI systems, installations, and users remain disproportionately concentrated in high-income countries, hampering the effective delivery of healthcare in LMICs. The diversity and intricacy of MRI data generated at such locations further complicate the transport, sharing, and joint analysis of such information for collaborative clinical decision support or machine-learning model training. Many LMICs are already investing in the expansion of MRI services and, therefore, methods to accelerate service establishment without imposing additional infrastructure or equipment needs retain substantial appeal. High-dimensional data obtained concurrently from Multiple-Input Multiple-Output (MIMO) Radio-frequency-coils help intensify operational significance in such layouts (34, 35). The collaborative medical improvement facilitated by concentrated data acquisition at the country level and uncomplicated processing procedures for understandability underpin the analysis of Correlation-MRI datasets and corresponding techniques (36).

### 6.4 Collaboration, data sharing, and governance

Numerous collaborative, multidisciplinary programs have made neuroimaging datasets available to a broad range of researchers and opened the possibility of jointly proposing models and algorithms. The variety of brain diseases, experimental protocols, and methodologies captured in these datasets is great, much wider than typically encountered in a local lab. Furthermore, the complexity of these systems and the multiscale nature of the relevant data demand expertise in many fields, a challenge often best addressed through teamwork (37). An infrastructure for sharing neuroimaging datasets, even in just a single modality, is therefore a key element that facilitates both the analysis of the data and the broadened understanding of a wide range of complex problems. Such an infrastructure embraces a common agreement on all aspects of the research, from the experimental design and image acquisition protocols to the tools used for pre-processing and analysis. The interaction among diverse teams is encouraged through conferences and workshops. Distribution of datasets is via a web portal that enables documentation, version tracking, and easy downloads while ensuring remote accessibility (38). Standardization of data formats, scanning protocols, image pre-processing routines, and related items extends the life of free image databases and enhances their potential for wider access and increased usage.

### 6.5 Policy and regulatory considerations

Despite important advancements in MR imaging, implementation across low- and middle-income countries (LMICs) remains very limited, and clinical access is highly inequitable. As of 2016, nearly 70% of worldwide MRI units were installed in high-income countries (HICs) which accounted for only 15% of the global population while 50 LMICs had no access to MR imaging and another 88 had only a single unit (39, 40). MRI installation patterns are also strongly influenced by technology, healthcare infrastructure, geopolitical factors, funding schemes, and socioeconomic conditions, with a very high prevalence of low field systems in many low resource nations.

Similarly, MRI representative and free access dataset availability remains an unresolved issue in the field that seriously compromises technological development and the construction of reliable models (3, 17) that may contribute to the significant improvement of medical imaging algorithms in diverse low-resource environments. Consequently, the compelling need to develop imaging methods that allow maximum acquisition of medically relevant information has led to the exploration of an extended acquired multidimensional representation for MR processes of unprecedented generality, supported by dedicated approaches that include the simultaneous acquisition of multiple-dimensional data in joint experiments.

## 7 Case studies and emerging evidence

Access to magnetic resonance imaging (MRI) remains limited in many low- and middle-income countries (LMICs), constraining efforts to initiate or bolster the use of this vital imaging modality. A sizeable number of LMICs have either no MRI capabilities or are restricted to only one or two facilities. Even where MRI is available, substantial logistical hurdles remain, including a lack of personnel trained in MRI techniques (3). McMRI, in limited clinical settings, has the potential to accelerate scanner acquisition times while maintaining important clinical specifications (41). Utilization of MC-MRI has also been proposed as a means to integrate multiple modalities into a common framework. The motivation for these developments is well established; experience to date suggests that many opportunities remain on the computational side.

Several emerging approaches have been developed to address MRI access challenges in LMICs and to maximize the clinical value of multidimensional imaging techniques. These include work on ultra-low-field portable scanners, the application of methods applicable to low-field or even zero-field MRI (pMRI) in LMIC settings a neuronavigation system and a portable positron emission tomography scanner, and broader studies of low-field and portable MRI. A major objective is to organize and translate these emerging experiences into practical implementation strategies that can be adopted by other LMICs facing similar healthcare constraints.

Considerable interest has arisen, primarily during the pandemic, in approaches for the accelerated treatment and acquisition of multi-contrast images, fully concurrent sequence blending, manifold-matching of multi-spectral/timed data, and the associated design of temporal and multi-spectral scenes designed to convey high-quality information via minimal sampling. Addressing these opportunities represents a forward-looking as well as LMIC-oriented strategy within a larger, structured initiative.

Several ongoing evaluations of sharedaccess initiatives to enhance diagnostic capacity in underserved LMICs have demonstrated measurable improvements in diagnostic confidence, clinical reporting accuracy, and interpretation reliability at the pilot stage. Studies of shared-access diagnostic models and low-cost imaging strategies confirm meaningful opportunities for expanding McMRI access in underserved regions. Notably, early deployment of ultra-low-field portable MRI (pMRI) for brain tumour and neurological evaluation in LMICs has established proof-of-concept for multi-contrast data acquisition at low field strengths, demonstrating that clinically actionable information can be obtained even in environments without access to high-field scanners (41).

### 7.1 Demonstrated benefits in clinical scenarios

**Figure 4:**
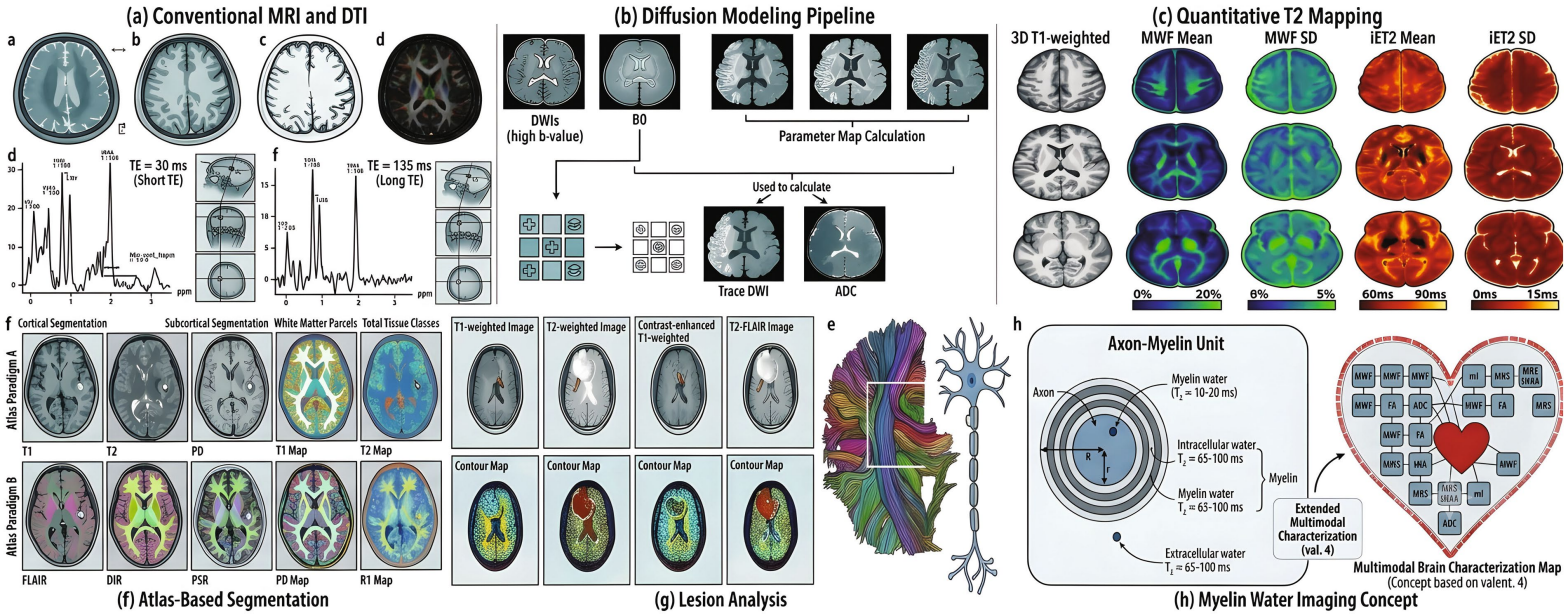
This figure shows how multidimensional maps improve differentiation between tumors, abscesses, and glioma grades. This demonstrate diagnostic gains, linking visual evidence with reported sensitivity/specificity improvements from prior studies (57). (Adapted from cited sources; see (42, 57)).

Multidimensional correlation MRI leverages time- or frequency-dependent relationships among multiscale data acquired across diverse physical modalities to facilitate the structure-aware joint acquisition, reconstruction, and interpretation of heterogeneous yet interrelated datasets. The period and spectrum of a physiological signal such as cardiac pulsation or respiration markedly influences all the recorded modalities, enabling a more efficient joint separation of the underlying sources. The practical combination of electromagnetic and sound waves in electromagnetic acoustic imaging, and the simultaneous measurement of the magnetic field and circuit current in denoising of magnetic-source measurements are among the other examples in which interdisciplinary correlation is exploited (42) (see Figure 4).

McMRI improves image quality and diagnostic accuracy by integrating information from multiple physical and temporal dimensions simultaneously, enabling richer tissue characterisation and more reliable reconstruction of complex clinical data (43). By exploiting correlations between signal dimensions such as T1/T2 relaxation, diffusion, and spectral profiles, McMRI allows clinicians to detect subtle pathological changes that single-parameter methods may miss. This integrative approach supports more precise clinical decision-making and opens new opportunities for computational innovation in resource-constrained settings.

### 7.2 Lessons from pilot programs and trials

Multidimensional MRI frameworks offer a promising solution by reducing acquisition complexity while preserving clinically relevant information. These frameworks enable simultaneous analysis of multiple signal parameters, improving noise suppression and image resolution, and allowing detection of subtle tissue changes that single-parameter methods may miss (44, 45).

Recent pilot studies have shown that such approaches can enhance diagnostic accuracy and expand patient access to advanced imaging (46). Implementation involved standardized parameter processing, resource allocation, flow analysis, and protocol adjustments to ensure quality control. Diverse equipment configurations and structured training programs facilitated adoption, while governance models supported sustainable integration. Early access to domain knowledge and collaborative networks proved critical for building capacity. Successful pilot programmes have demonstrated the importance of aligning local research priorities with global collaboration frameworks. Embedding McMRI capacity-building within existing LMIC health system structures, rather than introducing parallel infrastructure, has proven critical to sustainable adoption. Shared governance, open data agreements, and international partnerships that transfer both technology and training represent the most replicable pathways for expanding McMRI implementation across LMIC contexts (46).

### 7.3 Privacy, governance, and data sovereignty in LMIC portable MRI

The deployment of portable MRI in low-resource environments introduces unique ethical, legal, and social challenges that extend beyond conventional hospital-based imaging. Privacy concerns are particularly acute when scans are performed in community or bedside settings, where patient confidentiality is harder to guarantee. Informed consent procedures may be inconsistent, especially in mobile or informal contexts, and the absence of secure digital consent records increases the risk of data misuse. Encryption, anonymization, and secure transmission protocols are therefore essential safeguards, but their implementation is often constrained by limited technical infrastructure in LMICs (44).

Equally pressing are governance challenges. Portable MRI systems deployed outside institutional environments—such as ambulances, community centers, or even public spaces—operate beyond the reach of standard institutional review board (IRB) oversight. This creates regulatory gaps in accountability, particularly when devices are managed by non-professional operators or citizen scientists. LMICs require portable-specific governance frameworks that clarify responsibility for device operation, data management, and patient safety (44). Case examples from Nigeria highlight pilot programs where portable MRI governance is being tested, while Uganda’s telemedicine initiatives demonstrate how ethical safeguards can be integrated into imaging workflows under local regulatory supervision.

Finally, data sovereignty is a critical dimension of ethical deployment. Imaging datasets collected in LMICs must remain under local control to strengthen domestic healthcare systems and research capacity. Exporting data to external servers without oversight risks creating dependency and undermining national sovereignty. Hybrid cloud solutions, data trusts, and co-governance agreements offer potential pathways to balance accessibility with sovereignty, ensuring that imaging data serves local priorities while enabling international collaboration.

Together, these considerations underscore the need for privacy safeguards, governance frameworks, and data sovereignty policies tailored to LMIC contexts. Building regulatory capacity, training personnel, and embedding ethical oversight into portable MRI programs will be essential to ensure that technological innovation translates into equitable and sustainable healthcare outcomes.

### 7.4 Reproducibility, metadata standards, and open-source pipelines

Ensuring reproducibility in McMRI research and clinical deployment requires adoption of standardised data formats, metadata schemas, and openly validated reconstruction pipelines. For LMIC contexts, where institutional infrastructure varies considerably, adherence to widely accepted open standards is particularly critical to enable data sharing, multi-site validation, and interoperability across scanner platforms.

Image data should be stored and shared in DICOM (Digital Imaging and Communications in Medicine) format, which is the international standard for medical imaging and is supported by all major MRI scanner manufacturers (47). For research-grade McMRI datasets, the Brain Imaging Data Structure (BIDS) framework provides a standardised directory and metadata convention that facilitates reproducibility, automated processing, and open data publication. BIDS extensions for quantitative MRI and spectroscopy are directly applicable to McMRI workflows and are recommended for adoption in LMIC research programmes (48, 49).

Minimum metadata requirements for reproducible McMRI acquisitions should include: scanner manufacturer, field strength, and software version; pulse sequence parameters (TR, TE, flip angle, bandwidth); patient positioning and coil configuration; and any post-processing algorithms applied with version numbers. Incomplete metadata is a primary obstacle to cross-site validation and should be addressed through institutional data governance policies.

Open-source reconstruction pipelines are essential to make McMRI accessible in resource-constrained environments. MRzero (50) (an open, differentiable MRI simulator and sequence optimisation tool) and BART (Berkeley Advanced Reconstruction Toolbox) (51) are particularly relevant: BART provides a comprehensive open-source framework for iterative and compressed-sensing MRI reconstruction that can be deployed on modest hardware, making it well suited to LMIC computational environments. Both tools support community-driven validation and peer review of reconstruction algorithms, in line with best practices for algorithmic transparency recommended in Section 5.3. LMIC institutions are encouraged to adopt and contribute to these open-source ecosystems as a practical strategy for building local reconstruction expertise without proprietary licensing costs (18).

## 8 Future directions and research priorities

**Figure 5:**
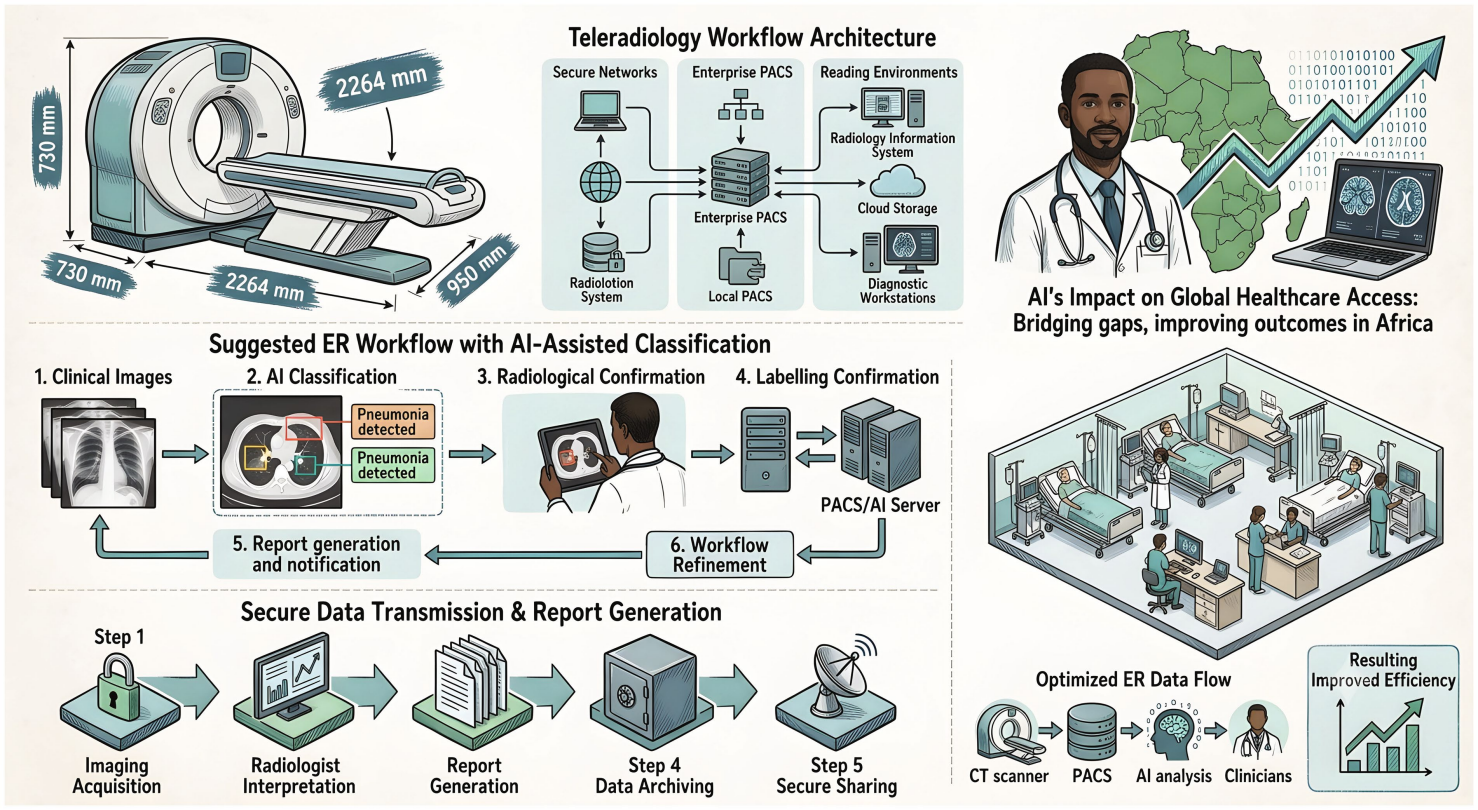
This figure illustrates how MC-MRI supports remote interpretation and telemedicine workflows. It is tied into the LMIC deployment discussion, showing how multidimensional datasets can be transmitted to distant diagnostic centers, thereby overcoming local expertise shortages and enabling collaborative healthcare delivery (58).

Healthcare challenges vary considerably between countries that possess similar economic status and GDP per capita, therefore disease burden and resulting prioritization of MR are important factors to consider when identifying specific needs. Disease burden and prioritization of diagnostic tools to address these needs play a key role in determining the necessity and utility of developing multidimensional correlation MRI and the underlying computational techniques in low- and middle-income countries (see Figure 5).

Tuberculosis remains an endemic condition in many low- and middle-income countries and contributes significantly to disability-adjusted life years (DALYs), highlighting the need for improved diagnostic imaging tools (52). Within this context, the development of multidimensional correlation MRI (McMRI) gains particular relevance, guiding the selection of imaging modalities such as 3D time of flight arterial spin labeling and 3D unenhanced contrast enhanced MRI. These techniques are favored over alternatives including positron emission tomography, functional MRI, and magnetoencephalography, given their suitability for addressing diagnostic challenges in resource-constrained environments. Scans can be completed in under 10 min and are compatible with low-field-strength systems (41), enabling the remote imaging techniques supported by low-field MRI systems can improve diagnostic access in areas where radiologists and oncology specialists are not readily available. MRI, MR diffusion model, and various other imaging disciplines have been fused with ultrasound techniques that accelerate the data-acquisition step of these diagnostic processes.

## 9 Conclusion

Multidimensional Correlation MRI (McMRI) has strong potential to improve diagnostic capacity in low- and middle-income countries (LMICs), especially for high-burden diseases such as tuberculosis. Its promise lies in reducing delays, improving diagnostic confidence, and modernizing limited MRI infrastructures. Advances in computational imaging methods, multimodal integration, and telemedicine can further expand access to care in resource-constrained settings. Recent work on advanced imaging frameworks (52) and efficient computational imaging pipelines (53) underscores the feasibility of scalable, integrated approaches. The successful translation of McMRI into LMIC practice will depend on aligning technical innovation with equity, embedding privacy safeguards, governance frameworks, and data sovereignty policies into deployment strategies. In essence, McMRI offers not only a diagnostic innovation but also a model for equitable technological integration in global health.

## References

[1] Qin C Murali S Lee E Supramaniam V Hausenloy DJ Obungoloch J . Sustainable low-field cardiovascular magnetic resonance in changing healthcare systems. Eur. Heart J. Cardiovasc. Imaging. (2022) 23:e246–60. doi: 10.1093/ehjci/jeab286, 35157038 PMC9159744

[2] Murali S Ding H Adedeji F Qin C Obungoloch J Sirkis T . Bringing MRI to low-and middle-income countries: directions, challenges and potential solutions. NMR Biomed. (2024) 37:e4992. doi: 10.1002/nbm.4992 37401341

[3] Jalloul M Miranda-Schaeubinger M Noor AM Stein JM Amiruddin R Derbew HM . MRI scarcity in low-and middle-income countries. NMR Biomed. (2023) 36:e5022. doi: 10.1002/nbm.5022, 37574441

[4] Thenuwara G Curtin J Tian F. Advances in diagnostic tools and therapeutic approaches for gliomas: a comprehensive review. Sensors. (2023) 23:9842. doi: 10.3390/s23249842, 38139688 PMC10747598

[5] Zhou H. Zhou F. Zhao C. Xu Y. Luo L. Chen H. (2024). Multimodal data integration for precision oncology: challenges and future directions. arXiv preprint arXiv:2406.19611.

[6] Avram AV Sarlls JE Basser PJ. Whole-brain imaging of subvoxel T1-diffusion correlation spectra in human subjects. Front Neurosci. (2021) 15:671465. doi: 10.3389/fnins.2021.671465, 34177451 PMC8232058

[7] Shinkareva SV Wang J Wedell DH. Examining similarity structure: multidimensional scaling and related approaches in neuroimaging. Comput Math Methods Med. (2013) 2013:796183. doi: 10.1155/2013/796183, 23662162 PMC3639644

[8] Fang F Wang T Zhang G Li F. Digging deeper in gradient for unrolling-based accelerated MRI reconstruction. IEEE Trans Pattern Anal Mach Intell. (2025) 47:4156–69. doi: 10.1109/TPAMI.2025.3540218, 40031599

[9] Kociuba MC Rowe DB. Complex-valued time-series correlation increases sensitivity in fMRI analysis. Magn Reson Imaging. (2016) 34:765–70. doi: 10.1016/j.mri.2016.03.011, 26988705

[10] Bruno F Arrigoni F Mariani S Splendiani A Di Cesare E Masciocchi C . Advanced magnetic resonance imaging (MRI) of soft tissue tumors: techniques and applications. Radiol Med. (2019) 124:243–52. doi: 10.1007/s11547-019-01035-7, 30949892

[11] Guyader JM Huizinga W Poot DH van Kranenburg M Uitterdijk A Niessen WJ . Groupwise image registration based on a total correlation dissimilarity measure for quantitative MRI and dynamic imaging data. Sci Rep. (2018) 8:13112. doi: 10.1038/s41598-018-31474-7 30166626 PMC6117310

[12] Cichocki A Mandic D De Lathauwer L Zhou G Zhao Q Caiafa C . Tensor decompositions for signal processing applications: from two-way to multiway component analysis. IEEE Signal Process Mag. (2015) 32:145–63. doi: 10.1109/msp.2013.2297439

[13] Beckmann CF Smith SM. Probabilistic independent component analysis for functional magnetic resonance imaging. IEEE Trans Med Imaging. (2004) 23:137–52. doi: 10.1109/TMI.2003.822821, 14964560

[14] Hilabi BS Alghamdi SA Almanaa M Alghamdi SA Sr. Impact of magnetic resonance imaging on healthcare in low-and middle-income countries. Cureus. (2023) 15:e37698. doi: 10.7759/cureus.37698, 37081900 PMC10112545

[15] Anazodo UC Ng JJ Ehiogu B Obungoloch J Fatade A Mutsaerts HJ . A framework for advancing sustainable MRI access in Africa. medRxiv. (2022) 10.1002/nbm.4846 36259628

[16] Frija G Blažić I Frush DP Hierath M Kawooya M Donoso-Bach L . How to improve access to medical imaging in low-and middle-income countries? EClinicalMedicine. (2021) 38:101034. doi: 10.1016/j.eclinm.2021.101034, 34337368 PMC8318869

[17] Shem S Ugwu AC Hamidu AU Flavious N Ibrahim M Zira D. Challenges, opportunities and strategies of global health radiology in low and middle-income countries (LMICs): an excerpt review. J Cancer Prev Curr Res. (2022) 13:14–20. doi: 10.15406/jcpcr.2022.13.00480

[18] Ahishakiye E Van Gijzen MB Tumwiine J Obungoloch J. Adaptive-size dictionary learning using information theoretic criteria for image reconstruction from undersampled k-space data in low field magnetic resonance imaging. BMC Med Imaging. (2020) 20:72. doi: 10.1186/s12880-020-00474-3, 32600272 PMC7477908

[19] Marey A Ambrozaite O Afifi A Agarwal R Chellappa R Adeleke S . A perspective on AI implementation in medical imaging in LMICs: challenges, priorities, and strategies. Eur Radiol. (2026) 36:2591–602. doi: 10.1007/s00330-025-12031-z, 41128758 PMC13035529

[20] Jones DK Alexander DC Chetcuti K Cercignani M Donald KA Griswold MA . Low field, high impact: democratizing MRI for clinical and research innovation. BJR| Open. (2025) 7:tzaf022. doi: 10.1093/bjro/tzaf022, 41112323 PMC12529269

[21] Khanh Nguyen Thi Bich H Bette S Hamm B Bauner KU. Multiparametric MRI: from simultaneous rapid acquisition methods and analysis techniques using scoring, machine learning, radiomics, and deep learning to the generation of novel metrics. Investig Radiol. (2023) 58:548–60. doi: 10.1097/RLI.0000000000000962, 36822661 PMC10332659

[22] Menacho K Ramirez S Segura P Nordin S Abdel-Gadir A Illatopa V . INCA (Peru) study: impact of non-invasive cardiac magnetic resonance assessment in the developing world. J Am Heart Assoc. (2018) 7:e008981. doi: 10.1161/JAHA.118.008981, 30371164 PMC6201420

[23] Zhao Z Wu J Li T Sun C Yan R Chen X. Challenges and opportunities of AI-enabled monitoring, diagnosis & prognosis: a review. Chin J Mech Eng. (2021) 34:56. doi: 10.1186/s10033-021-00570-7

[24] Koirala N Kleinman D Perdue MV Su X Villa M Grigorenko EL . Widespread effects of dMRI data quality on diffusion measures in children. Hum Brain Mapp. (2022) 43:1326–41. doi: 10.1002/hbm.25724, 34799957 PMC8837592

[25] Yang G Jan MA Rehman AU Babar M Aimal MM Verma S. Interoperability and data storage in internet of multimedia things: investigating current trends, research challenges and future directions. IEEE Access. (2020) 8:124382–401. doi: 10.1109/access.2020.3006036

[26] Ouedraogo EB Hawbani A Wang X Liu Z Zhao L Al-qaness MA . Digital twin data management: a comprehensive review. IEEE Trans Big Data. (2025) 11:2224–43. doi: 10.1109/tbdata.2025.3533891

[27] Peixoto T Oliveira Ó Costa e Silva E Oliveira B Ribeiro F. A data quality pipeline for industrial environments: architecture and implementation. Comput. (2025) 14:241. doi: 10.3390/computers14070241

[28] Bernabei JM Sinha N Arnold TC Conrad E Ong I Pattnaik AR . Normative intracranial EEG maps epileptogenic tissues in focal epilepsy. Brain. (2022) 145:1949–61. doi: 10.1093/brain/awab480, 35640886 PMC9630716

[29] Mollura DJ Culp MP Pollack E Battino G Scheel JR Mango VL . Artificial intelligence in low-and middle-income countries: innovating global health radiology. Radiology. (2020) 297:513–20. doi: 10.1148/radiol.2020201434, 33021895

[30] Yamashita A Yahata N Itahashi T Lisi G Yamada T Ichikawa N . Harmonization of resting-state functional MRI data across multiple imaging sites via the separation of site differences into sampling bias and measurement bias. PLoS Biol. (2019) 17:e3000042. doi: 10.1371/journal.pbio.3000042, 30998673 PMC6472734

[31] Ohannessian R Duong TA Odone A. Global telemedicine implementation and integration within health systems to fight the COVID-19 pandemic: a call to action. JMIR Public Health Surveill. (2020) 6:e18810. doi: 10.2196/18810, 32238336 PMC7124951

[32] Abhisheka B Biswas SK Purkayastha B Das D Escargueil A. Recent trend in medical imaging modalities and their applications in disease diagnosis: a review. Multim Tools Appl. (2024) 83:43035–70. doi: 10.1007/s11042-023-17326-1

[33] Louie A. Multimodality imaging probes: design and challenges. Chem Rev. (2010) 110:3146–95. doi: 10.1021/cr9003538, 20225900 PMC2878382

[34] Menet N Hersche M Karunaratne G Benini L Sebastian A Rahimi A. Mimonets: multiple-input-multiple-output neural networks exploiting computation in superposition. Adv Neural Inf Proces Syst. (2023) 36:39553–65.

[35] López-Bueno D Montoro G Gilabert PL. Training data selection and dimensionality reduction for polynomial and artificial neural network MIMO adaptive digital predistortion. IEEE Trans Microw Theory Tech. (2022) 70:4940–54. doi: 10.1109/tmtt.2022.3209214

[36] Keenan KE Ainslie M Barker AJ Boss MA Cecil KM Charles C . Recommendations toward standards for quantitative MRI (qMRI) and outstanding needs. J Magn Reson Imaging. (2021) e26–e39. doi: 10.1002/jmri.26598 PMC6663309 30680836

[37] Scott A Courtney W Wood D De la Garza R Lane S King M . COINS: an innovative informatics and neuroimaging tool suite built for large heterogeneous datasets. Front Neuroinform. (2011) 5:33. doi: 10.3389/fninf.2011.00033 22275896 PMC3250631

[38] Poline JB Breeze JL Ghosh S Gorgolewski K Halchenko YO Hanke M . Data sharing in neuroimaging research. Front Neuroinform. (2012) 6:9. doi: 10.3389/fninf.2012.00009, 22493576 PMC3319918

[39] Arnold TC Freeman CW Litt B Stein JM. Low-field MRI: clinical promise and challenges. J Magn Reson Imaging. (2023) 57:25–44. doi: 10.1002/jmri.28408, 36120962 PMC9771987

[40] Wilson CJ. An Exploratory Needs Assessment for Medical Devices in Low-and Middle-Income Countries [dissertation on the Internet]. Villanova: Villanova University (2022). Available online at: https://www.proquest.com/openview/c678be8fb29775e72653a72e7cd1dd4d/1?pq-origsite=gscholar&cbl=18750&diss=y (Accessed July 6, 2026).

[41] Altaf A Baqai MWS Urooj F Alam MS Aziz HF Mubarak F . Utilization of an ultra-low-field, portable magnetic resonance imaging for brain tumor assessment in lower middle-income countries. Surg Neurol Int. (2023) 14:260. doi: 10.25259/SNI_123_2023, 37560587 PMC10408621

[42] Fang Z Gao F Jin H Liu S Wang W Zhang R . A review of emerging electromagnetic-acoustic sensing techniques for healthcare monitoring. IEEE Trans. Biomed. Circuits Syst. (2022) 16:1075–94. doi: 10.1109/TBCAS.2022.3226290, 36459601

[43] Lin W. Principles of magnetic resonance imaging: a signal processing perspective [book review]. IEEE Eng Med Biol Mag. (2000) 19:129–30. doi: 10.1109/MEMB.2000.870245, 25079929

[44] Shen FX Wolf SM Lawrenz F Comeau DS Dzirasa K Evans BJ . Ethical, legal, and policy challenges in field-based neuroimaging research using emerging portable MRI technologies: guidance for investigators and for oversight. J Law Biosci. (2024) 11:lsae008. doi: 10.1093/jlb/lsae008 38855036 PMC11157461

[45] Andreou C Weissleder R Kircher MF. Multiplexed imaging in oncology. Nat. Biomed. Eng. (2022) 6:527–40. doi: 10.1038/s41551-022-00891-5, 35624151

[46] Khalifa M Albadawy M. AI in diagnostic imaging: revolutionising accuracy and efficiency. Comput Methods Programs Biomed Update. (2024) 5:100146. doi: 10.1016/j.cmpbup.2024.100146

[47] Mildenberger P Eichelberg M Martin E. Introduction to the DICOM standard. Eur Radiol. (2002) 12:920–7. doi: 10.1007/s003300101100, 11960249

[48] Gorgolewski KJ Auer T Calhoun VD Craddock RC Das S Duff EP . The brain imaging data structure, a format for organizing and describing outputs of neuroimaging experiments. Sci Data. (2016) 3:160044. doi: 10.1038/sdata.2016.44, 27326542 PMC4978148

[49] Karakuzu A Appelhoff S Auer T Boudreau M Feingold F Khan AR . qMRI-BIDS: an extension to the brain imaging data structure for quantitative magnetic resonance imaging data. Sci Data. (2022) 9:517. doi: 10.1038/s41597-022-01571-4, 36002444 PMC9402561

[50] Loktyushin A Herz K Dang N Glang F Deshmane A Weinmüller S . MRzero – automated discovery of MRI sequences using supervised learning. Magn Reson Med. (2021) 86:709–24. doi: 10.1002/mrm.28727, 33755247

[51] Uecker M Ong F Tamir JI Bahri D Virtue P Cheng JY . Berkeley advanced reconstruction toolbox. Proc Intl Soc Mag Reson Med. (2015) 23:9. Available online at: https://users.ece.utexas.edu/~jtamir/files/papers/2486.pdf

[52] Alene KA Wangdi K Colquhoun S Chani K Islam T Rahevar K . Tuberculosis related disability: a systematic review and meta-analysis. BMC Med. (2021) 19:203. doi: 10.1186/s12916-021-02063-9, 34496845 PMC8426113

[53] Hayat M Aramvith S. Superpixel-guided graph-attention boundary GAN for adaptive feature refinement in scribble-supervised medical image segmentation. IEEE Access. (2025) 13:196654–68. doi: 10.1109/access.2025.3634156

[54] Saltarelli G Di Cerbo G Innocenzi A De Felici C Splendiani A Di Cesare E. Quantitative MRI in neuroimaging: a review of techniques, biomarkers, and emerging clinical applications. Brain Sci. (2025) 15:1088. doi: 10.3390/brainsci15101088, 41154182 PMC12562573

[55] Bi ZM Wang L. Advances in 3D data acquisition and processing for industrial applications. Robot Comput Integr Manuf. (2010) 26:403–13. doi: 10.1016/j.rcim.2010.03.003

[56] World Health Organization. Global Report on Infection Prevention and Control 2024. Cham: World Health Organization (2024).

[57] Whittall KP MacKay AL. Quantitative interpretation of NMR relaxation data. J. Magn. Reson. (1989) 84:134–52. doi: 10.1016/0022-2364(89)90011-5

[58] Topol E. Deep medicine: how artificial Intelligence can make Healthcare human again. 1st ed. New York: Basic Books (2019). p. 378.

[59] Hayat M Dhaliwal A Din MM Izhar R Nadeem M Ahmad N. Cross-attention patch fusion for few-shot colorectal tissue generation. In2025 5th International Conference on Digital Futures and Transformative Technologies (ICoDT2). (2025) (pp. 1–6). IEEE. doi: 10.1109/ICoDT269104.2025.11360745

[60] Ogbole GI Adeleye AO Adeyinka AO Ogunseyinde OA. Magnetic resonance imaging: clinical experience with an open low-field-strength scanner in a resource challenged African state. J Neurosci Rural Pract. (2012) 3:137–43. doi: 10.4103/0976-3147.98210 22865963 PMC3409982

[61] Radio Nigeria. Need to close gaps in Healthcare Diagnostics and power Supply. Radio Nigeria. (2026). Available online at: https://radionigeria.gov.ng/2026/02/28/need-to-close-gaps-in-healthcare-diagnostics-and-power-supply/

[62] Ojuroungbe S. MRI Machines in public Hospitals not Working due to erratic power Supply, Says Resident Doctors. PUNCH Healthwise. (2025). Available online at: https://healthwise.punchng.com

[63] Tribune Online. FG Deploys 1.5MW solar power system to Kano Orthopaedic Hospital. Tribune Online. (2025). Available online at: https://tribuneonlineng.com/fg-deploys-1-5mw-solar-power-system-to-kano-orthopaedic-hospital/

[64] Bulamu A Muyinda Z Nalwanga F . A translational research leveraging diagnostic accuracy of innovations in MRI as a model for early breast cancer detection in Uganda. Technol Cancer Res Treat. (2025) 24:15330338251356549. doi: 10.1177/15330338251356549 40660819 PMC12264417

[65] Gateway News UG. BIG SCORE: Kisubi Hospital Acquires East Africa’s first next-Generation MRI Scanner. Gateway News UG. (2025). Available online at: https://www.gatewaynews.co.ug/big-score-kisubi-hospital-acquires-east-africas-first-next-generation-mri-scanner/

[66] Shrinuvasan S Chidambaram R. Evaluation of low back pain with low field open magnetic resonance imaging scanner in rural hospital of southern India. J Neurosci Rural Pract. (2016) 7:368–73. 27365953 10.4103/0976-3147.181455 PMC4898104

[67] Pires T Jaseemudheen MM. The rise and efficiency of low field portable MRI scanners. Journal of Health and Allied Sciences NU. (2024) 14:163–8.

[68] Rathebe PC. Perceived safety of MRI units in the two public hospitals within the central region of South Africa: a pilot study among four MR staff. J Public Health Res. (2022) 11:22799036221123386. doi: 10.1177/22799036221123386 36185417 PMC9520159

[69] Rathebe PC. Subjective symptoms of SMFs and RF energy, and risk perception among staff working with MR scanners within two public hospitals in South Africa. Electromagn Biol Med. (2022) 41:152–62. doi: 10.1080/15368378.2022.2031212 35139718

